# Vulnerability index impacts distribution of prostate cancer clinical trials in the United States

**DOI:** 10.3389/fonc.2026.1874405

**Published:** 2026-10-05

**Authors:** Vitor Abreu de Goes, Benjamin Mercier, Yu Jun Li, Koral Shah, Xiaochen Li, George Zhang, Daniela V. Castro, Jadon Fann, Salvador Jaime-Casas, Lauren M. Lau, Lily M. Lau, Joshua Lee, Kyra Shah, Ethan Swarat, Akasha Dukkipati, Ruchi Agarwal, Ruth Simon, Jaya Goud, Rahul Winayak, Miguel Zugman, Regina Barragan-Carrillo, Nazli Dizman, Charles Nguyen, Sumanta K. Pal, Alexander Chehrazi-Raffle

**Affiliations:** 1 Department of Medical Oncology and Experimental Therapeutics, City of Hope Comprehensive Cancer Center, Duarte, CA, United States; 2 MD Anderson Cancer Center, Houston, TX, United States

**Keywords:** clinical trial, disparities, distribution, prostate cancer, United States, vulnerability

## Abstract

**Background:**

Availability of clinical trials for prostate cancer in the US varies greatly by region. County-level analyses are vital to characterize healthcare access, disease burden, and population-level disparities.

**Methods:**

Utilizing the ClinicalTrials.gov trial database, we collated all interventional prostate cancer trials between June 1, 2019, and June 1, 2025. We compiled data on county characteristics along with Social Vulnerability Index (SVI) designations derived from the CDC, NCI, and the Agency for Toxic Substances and Disease Registry data. A zero-inflated negative binomial regression model was used to evaluate trial availability. Trial characteristics were summarized using descriptive analysis.

**Results:**

We found 884 eligible trials. Among 3,145 counties, only 631 had at least one clinical trial over the study period. Most trials (51%) included patients with metastatic disease. Sixteen percent of trials investigated curative treatments, while 65% examined palliative interventions. The majority of studies (52.5%) had funding from pharmaceutical companies. There was no significant relationship between trial availability and incidence or mortality rates. Among counties with at least one trial, those with a low SVI had twice as many trials per household when compared to those with high SVI (IRR 2.05, 95% CI 1.54 - 2.74, p<0.001). Counties with no trials were more rural (72.3% vs 22.0%) and had fewer specialist physicians (17.1% vs 69.1%).

**Conclusion:**

Prostate cancer trials are disproportionately concentrated in the US, with lower representation in vulnerable areas. Expanding trial access to underserved areas is a crucial step in promoting equitable access to care.

## Introduction

Prostate cancer is the second leading cause of cancer-related deaths in the United States, accounting for approximately 15% of all new cancer cases in 2025 (1, 2). Clinical trials have been pivotal to improving patient outcomes in this disease space, including quality of life and survival rates (3–8). Access to clinical trials is thus paramount for developing better therapeutic strategies for patients with prostate cancer.

However, cancer clinical trials remain disproportionately concentrated in large academic centers near major metropolitan areas (2, 9–11). Nearly 55% of patients with a diagnosis of cancer have been shown to have inadequate access to clinical trials, which is thought to be largely driven by geographic barriers to care (2). Population-level social determinants of health have also been linked to worse clinical outcomes and reduced access to clinical trial opportunities (12–15).

County-level analyses provide a granular view of healthcare access, disease burden, and population-level disparities (16, 17). Thus, we examined the geographic distribution of prostate cancer clinical trials across U.S. counties and its relationship with social determinants of health and prostate cancer burden.

## Methods

Utilizing the ClinicalTrials.gov trial database, we compiled all interventional prostate cancer trials between June 1, 2019, and June 1, 2025. Studies evaluating screening/diagnosis have been deemed interventional. Trials were required to include site location information and at least one domestic trial site. Site locations were matched to U.S. counties (or county equivalents) using ZIP codes and available Postal Service data (18). Trials with sites in multiple counties were counted as a single unique trial per county, whereas those with multiple trial sites within the same county were counted once. In the rare instance where a ZIP code corresponded to more than one county, all associated counties were included as having that trial. Given administrative boundary changes in Connecticut, trial sites were assigned to the former county boundaries to align with available county-level data (19). Sites in Puerto Rico and Guam were retained in the analysis. Due to limitations in mapping them in ClinicalTrials.gov ZIP code data to county-equivalent jurisdictions, they were categorized at the territory level rather than assigned to specific counties.

In our analysis, we included trial sponsor, planned accrual goal, investigational phase, stage of disease, interventional goal, and county of each trial site. Multi-phase trials were classified according to the highest phase of the study. We assessed each county’s Social Vulnerability Index (SVI), a score based on 16 variables encompassing socioeconomic status, ethnic and racial minorities status, housing, and transportation. We also calculated age-adjusted prostate cancer incidence and mortality rates for each US county with available SVI data. Counties were stratified by prostate cancer incidence, mortality rate, and SVI, the latter of which was organized by High, Medium-high, Low-medium, and Low quartiles. These data were derived from the National Cancer Institute (NCI) and the Agency for Toxic Substances and Disease Registry (20, 21). Counties were also classified as rural or urban using rurality measures published by the U.S. Department of Agriculture (22). Physician workforce availability data were obtained from the Centers for Medicare & Medicaid Services (CMS) database (23). Those practicing in medical oncology, radiation oncology, and urology were identified by ZIP code and aggregated at the county level. Because physicians may be listed at multiple practice locations, each physician-location entry was counted separately.

### Statistical analysis

Trial characteristics were summarized using descriptive statistics. Due to the high number of countries with no clinical trials, we used a zero-inflated negative binomial (ZINB) regression model to accommodate the high dispersion of our dataset. This model adjusts for the high proportion of counties without trial sites and estimates potential influence of county characteristics on trial presence by stratifying those with and without trial sites into separate sub-analyses. The negative binomial model of the ZINB estimates how predictors, such as SVI or prostate cancer incidence, relate to the rate of trials per county household. Utilizing those same predictors, the zero-inflation model of the ZINB estimates how they relate to the probability of a county having zero trials within them. Incidence of prostate cancer trials are expressed as incidence rate ratios (IRRs) alongside their 95% confidence intervals (CI). Generation of the ZINB regression model was performed using the “pscl” package in R v4.4.3.

## Results

Our analysis yielded 884 eligible trials between 2019 and 2025. Among 3,145 counties, 631 (20.1%) had at least one clinical trial over the study period. The distribution of clinical trials across US counties is depicted in **Figure 1**.

**Figure 1:**
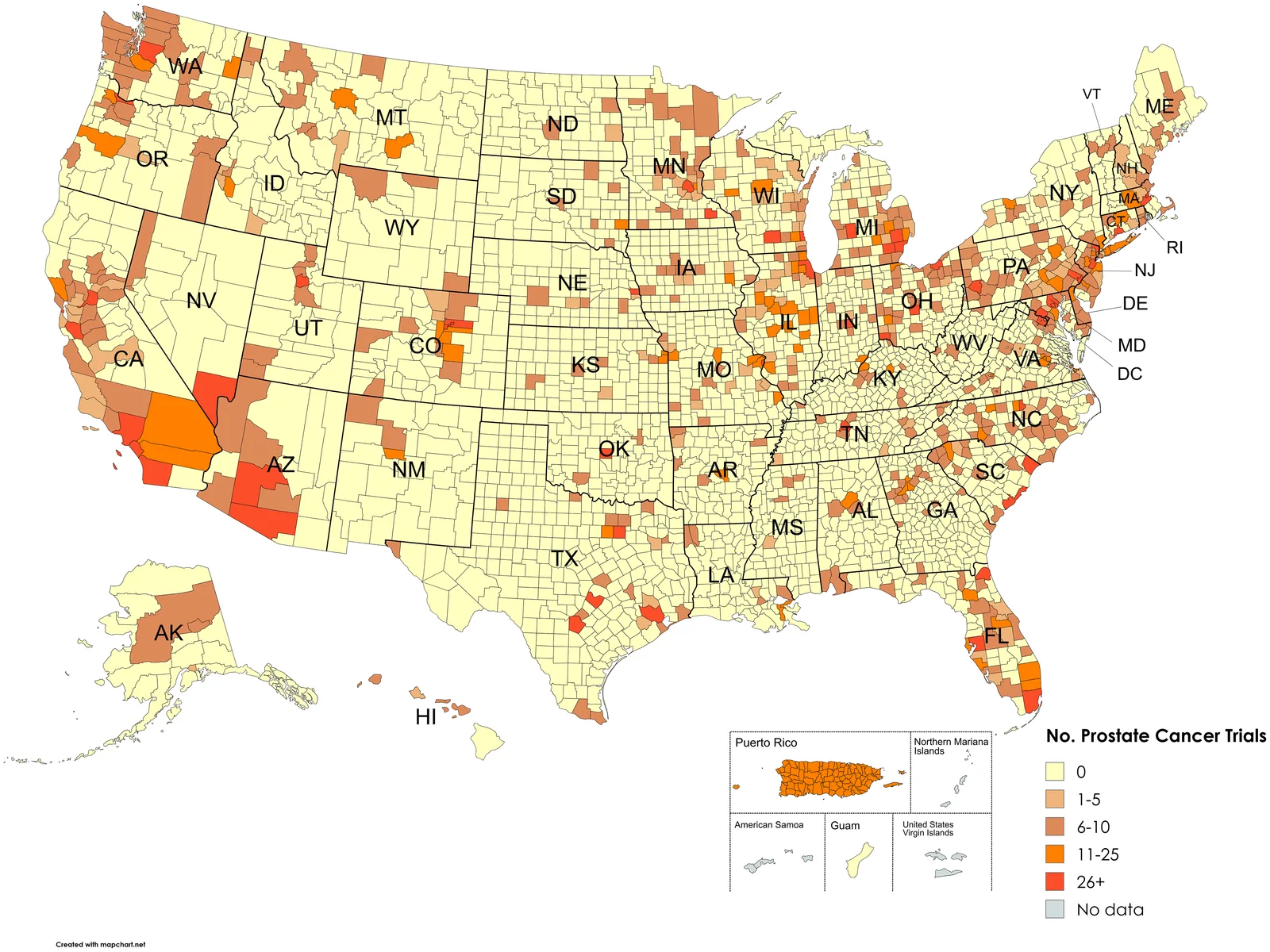
Distribution of prostate cancer clinical trials across U.S. counties. Trials with multiple sites within the same county were counted as a single trial. Created with mapchart.net.

### Trial characteristics

Most clinical trials (65.1%) were early-phase (Phase I/II), while 11.3% were late-phase (Phase III/IV), and 23.6% did not specify a study phase. The majority of trials had a median planned enrollment of fewer than 100 patients (62.7%), followed by trials with planned enrollment greater than 100 patients (33.7%), whereas 3.6% did not report planned enrollment. Most trials included patients with metastatic prostate cancer (60.4%), followed by localized disease (27.9%), while 11.7% did not specify any clinical stage. Studies were primarily sponsored by academia (60.6%), followed by pharmaceutical companies (34.8%) and governmental agencies (4.5%). More than half of the trials (52.5%) had some form of funding from pharmaceutical companies. Most trials (64.6%) investigated palliative interventions, 14.6% focused on diagnosis or screening, 15.9% on curative treatment, and 4.9% had mixed or other purposes. These findings are summarized in **Table 1**.

**Table 1:** Prostate cancer clinical trial characteristics.

Trial characteristics	Number of trials (N = 884)
Primary sponsor	
Academia (grant/university/institution)	536 (60.6%)
Government (NIH/NCI/Department of Justice)	40 (4.5%)
Pharmaceutical company	308 (34.8%)
Pharma funding	
Yes	464 (52.5%)
No	420 (47.5%)
Planned enrollment	
1–100	554 (62.7%)
>100	298 (33.7%)
Not available	32 (3.6%)
Trial phase	
Early phase (I, II)	575 (65.1%)
Late phase (III, IV)	100 (11.3%)
Not available	209 (23.6%)
Cancer stage	
Metastatic	534 (60.4%)
Non-Metastatic	247 (27.9%)
Not available	103 (11.7%)
Intervention Intention	
Curative	141 (15.9%)
Diagnostic/Screening	129 (14.6%)
Supportive care	571 (64.6%)
Other	43 (4.9%)

### Analysis by SVI quartiles

In the negative binomial component of the ZINB model, counties with a low SVI had approximately twice as many trials per household as those with a high SVI (IRR = 2.05, 95% CI: 1.54 - 2.74, *p* < 0.001). Counties with low-medium and medium-high SVI also had more than 60% more clinical trials per household (IRR = 1.63, 95% CI: 1.22 - 2.19; IRR = 1.61, 95% CI: 1.21 - 2.14, respectively; all *p* < 0.001). Prostate cancer incidence and mortality rates were comparable across all SVI quartiles (IRR = 1.00, 95% CI: 0.99 - 1.01, *p* = 0.16; IRR = 1.01, 95% CI: 0.98 - 1.03, *p* = 0.65). The results of the binomial component of the ZINB model are shown in **Table 2**. In the zero-inflation component of our model, counties with low-medium and low SVI had 2.9-fold (95% CI: 1.44 - 5.63) and 3.7-fold (95% CI: 1.56 - 8.78) higher odds of having no trials compared to high-SVI counties. The results of the zero-inflation component of the ZINB model are shown in **Table 3**.

**Table 2:** Negative binomial model evaluating the availability of prostate cancer trials by county characteristics.

Variable	Incidence rate ratio	95% CI	P-value
SVI (Low vs High)	2.05	(1.54 - 2.74)	<0.001
SVI (Low–Medium vs High)	1.63	(1.22 - 2.19)	0.0007
SVI (Medium–High vs High)	1.61	(1.21 - 2.14)	0.0009
Mortality per 100k	1.01	(0.98 - 1.03)	0.65
Incidence per 100k	1.00	(0.99 - 1.01)	0.16

This analysis includes only counties with at least one trial. For continuous variables, the incidence ratio was calculated per 1-unit increase. CI, confidence interval; SVI, social vulnerability index.

**Table 3:** Odds of a county having no clinical trials by social vulnerability index using a zero-inflated negative binomial regression.

Variable	Odds ratio	95% CI	P-value
SVI (Low vs High)	3.70	(1.56 - 8.78)	0.0028
SVI (Low–Medium vs High)	2.85	(1.44 - 5.63)	0.0027
SVI (Medium–High vs High)	1.57	(0.81 - 3.04)	0.19

CI, confidence interval; SVI, social vulnerability index.

Trials conducted in low-SVI counties were more likely to be sponsored by academia (61.7%)compared to low-medium (49.7%), medium-high (37.9%), and high-SVI counties (32.9%) (*p* < 0.001). In contrast, pharmaceutical sponsorship was more common in high-SVI counties (61.7%) compared to medium-high (57.0%), low-medium (43.4%), and low-SVI counties (30.6%) (*p* < 0.001). Trials in high-SVI counties often included metastatic patients (71.2% vs. 68.6%, 61.8%, and 52.6% for medium-high, low-medium, and low-SVI counties, respectively; *p* < 0.001). The subgroup analysis of trial characteristics per county SVI is shown in **Supplementary Table 1**.

### County characteristics

A total of 2,514 counties had no available clinical trials. Of these, 1,817 (72.3%) were classified as rural and 696 (27.7%) as urban. In contrast, among the 631 counties with at least one trial, 139 (22.0%) were rural and 492 (78.0%) were urban. Overall, 866 counties had at least one matching specialist physician (medical oncologist, radiation oncologist, or urologist). Among counties without available trials, 430 (17.1%) had a specialist physician, whereas 436 (69.1%) of counties with at least one trial had a relevant specialist. The mismatch between trial availability and the distribution of specialist physicians is illustrated in **Figure 2**.

**Figure 2:**
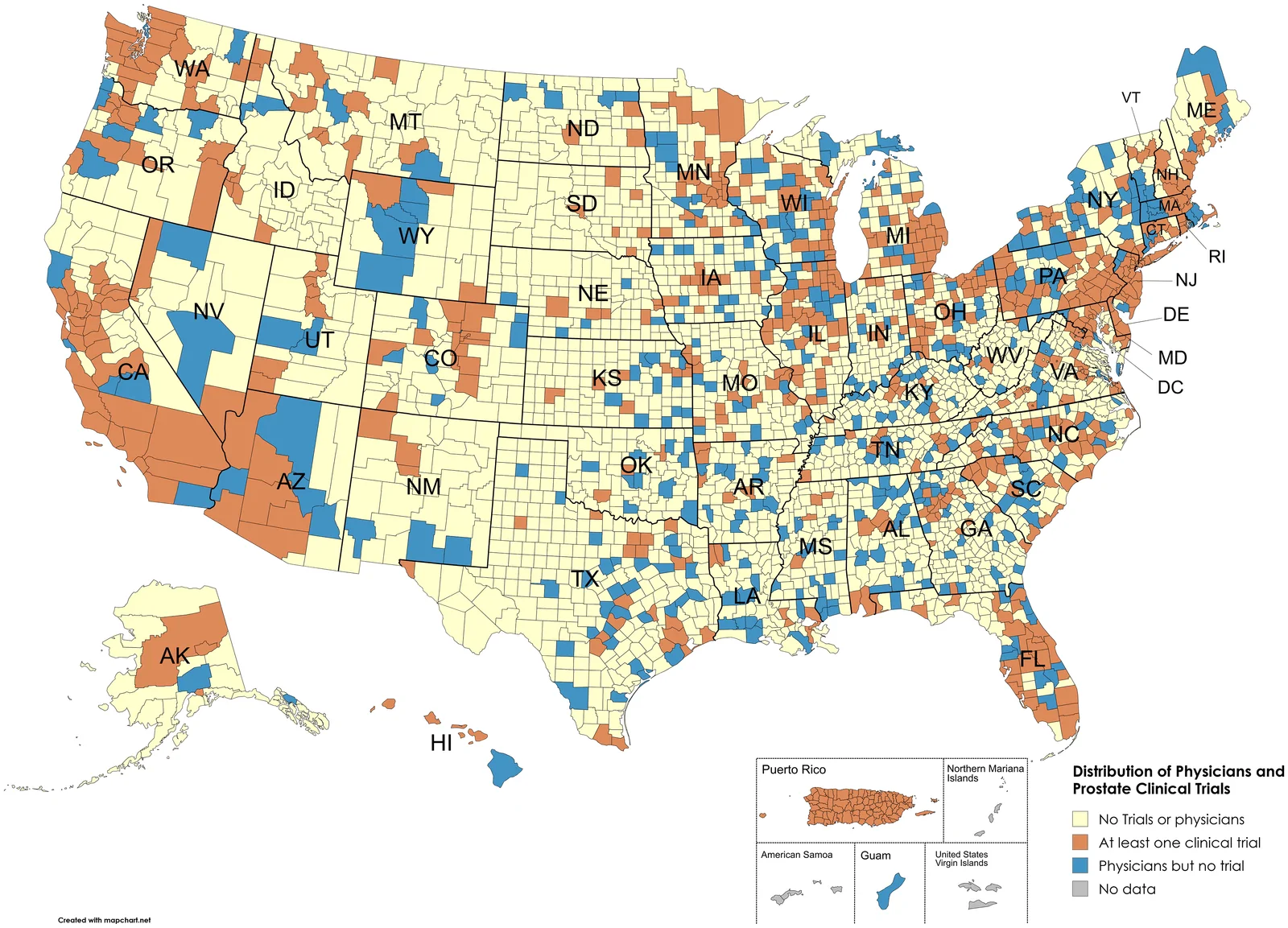
Distribution of prostate cancer trials versus specialized physicians (medical oncologists, radiation oncologists, and urologists) (2026). Data sources: ClinicalTrials.gov and Centers for Medicare & Medicaid Services (CMS) database. Created with mapchart.net.

## Discussion

The United States has the largest number of cancer clinical trials worldwide, yet their distribution within the country remains disproportionate (12, 24). In the study herein, nearly 80% of counties had no prostate cancer clinical trial sites. Among counties with at least one trial, those with a low SVI had twice as many trials as counties with a high SVI. This might be a reflection of impaired access to clinical trials among socially vulnerable communities, potentially widening disparities in prostate cancer care. However, low-SVI counties also had greater odds of having no trials, indicating that trial availability is influenced by multiple factors. Notably, counties with available trials were more urban and had more specialist physicians, consistent with prior reports. Prostate cancer incidence and mortality rates were not associated with trial availability.

The disparities reported in this study are concerning, as clinical trials are fundamental to the development of therapies that can improve upon the current prostate cancer treatment landscape. The underrepresentation of counties, only 631 of 3,145, means that most of the country lack firsthand access to emerging therapies. Moreover, because we did not include multiple sites for the same trial within each county, the actual trial concentration could be underreported, especially in counties with larger populations and/or land areas. The stepwise increase in trial counts as SVI decreases suggests that research activity is associated with socioeconomic factors, which has been previously described (17). This underlines barriers that affect communities already facing disparities in cancer care (15, 25, 26). A disproportionate distribution of trials could also lead to slower accrual and study populations that lack external representativeness. Of note, regional disparities in trial availability are not unique to prostate cancer, with similar trends observed across other malignancies, such as bladder, kidney, breast, colon, lung, pancreatic, and melanoma (26–30).

Interestingly, counties with low SVI also had greater odds of having no trials. However, this reflects a small absolute difference given the large number of counties without available trials and should be interpreted with caution. Overall, we understand the conjunct of our findings as indicating that higher SVI is associated with reduced trial availability, and differences between our results and previous reports likely stem from distinct analytic methodology (17). Nevertheless, the paradoxical relationship between SVI and trial availability suggests that additional factors also influence trial distribution. Accordingly, among counties without available trials, only 12.4% had at least one physician and most were classified as rural, both of which have been associated with reduced trial availability (31–35).

With this in mind, counties with practicing physicians but no trials could represent promising targets for expansion, as showcased in **Figure 2** (2, 35). Multicenter studies may be particularly attractive in this scenario, as they were associated with higher trial success rates and might help overcome local limitations in research infrastructure and leadership (35). Of note, we found that trials in high SVI counties tended enroll fewer patients and were more often funded by pharmaceutical companies, while academic sponsorship was more common at lower SVI counties, highlighting that sponsorship also plays a significant role in trial availability. The greater representation of industry-led studies in vulnerable counties suggests that they are better positioned to operate in this setting, and underscores the challenges faced by academic institutions to conduct research in underserved regions. Our results are consistent with existing literature, in which clinical trials’ reliance on academia has been reported across multiple malignancies (36–38).

Although pharma can be a valuable tool to expand access, distinct sponsorships have particular research questions and patient populations and should be viewed as complementary (39–41). Thus, a balanced portfolio is essential to ensure that research aligns with local population needs. Moreover, initiatives such as the NCI Community Oncology Research Program and the use of tools like the NCI Clinical Trials Assessment of Infrastructure Matrix and the Society for Clinical Research Sites Diversity Site Assessment Tool can help to bridge the gap by increasing research sites in vulnerable communities, provide scientific leadership, increase participant’s diversity and promote study designs that reflect local health priorities (42, 43). Finally, efforts like workforce training to reduce bias, and trial designs that reduce logistical and financial barriers may further improve participation among underserved populations (44).

This study has several limitations. As we did not collect the total number of sites for trials within the same county, our ability to exactly determine trial access, with variables such as driving time to nearest site, is diminished. Social vulnerability and health outcomes often vary widely within counties, therefore analysis of SVI at the county level could potentially average trial access disparities. In addition, SVI alone does not capture confounders that may impact trial availability and access, such as proximity to academic centers or the presence of NCI-designated centers. Clinical trial data came from a single registry, which could exclude trials listed elsewhere. Furthermore, this registry lacks standardization of trial characteristics, which may have introduced errors during manual extraction, especially in instances where recorded characteristics were incomplete.

## Conclusion

Prostate cancer trials are disproportionately concentrated in the U.S., and their distribution does not align with county-level incidence or mortality rates. Adequate characterization of social determinants of health is needed to guide expansion of clinical trial availability.

## Data Availability

The raw data supporting the conclusions of this article will be made available by the authors, without undue reservation.

## Supplementary material

Supplementary Table 1 Relationship between clinical trial characteristics and SVI quartiles using the total number of trial sites. P-values were calculated using chi-square test for categorical variables and the Kruskal–Wallis’s test for continuous variables.​ SVI, social vulnerability index.
