# Supplementary material for: Vulnerability index impacts distribution of prostate cancer clinical trials in the United States
Source: Front Oncol. 2026 Oct 5;16:1874405. doi: 10.3389/fonc.2026.1874405 (PMC13678669; doi:10.3389/fonc.2026.1874405)
Supplement: Supplementary file 1 [file Table1.docx]

**Supplementary Table 1.** Relationship between clinical trial characteristics and SVI quartiles using the total number of trial sites. P-values were calculated using chi-square test for categorical variables and the Kruskal–Wallis’s test for continuous variables. SVI, social vulnerability index.

|  | Number of unique trial sites (N=6840) | High SVI (N=2173) | Medium-high SVI (N=2150) | Low-medium SVI (N=1510) | Low SVI (N=1007) | P-value |
| --- | --- | --- | --- | --- | --- | --- |
| **Primary sponsor** |  |  |  |  |  | < 0.001 |
| Academia (grant/ university/ institution) | 2900 (42.4%) | 714 (32.9%) | 815 (37.9%) | 750 (49.7%) | 621 (61.7%) |  |
| Government (NIH/ NCI/ Department of Justice) | 411 (6.0%) | 119 (5.5%) | 110 (5.1%) | 104 (6.9%) | 78 (7.7%) |  |
| Pharmaceutical company | 3529 (51.6%) | 1340 (61.7%) | 1225 (57.0%) | 656 (43.4%) | 308 (30.6%) |  |
| **Pharma funding** |  |  |  |  |  | < 0.001 |
| No | 2990 (43.7%) | 715 (32.9%) | 836 (38.9%) | 761 (50.4%) | 678 (67.3%) |  |
| Yes | 3850 (56.3%) | 1458 (67.1%) | 1314 (61.1%) | 749 (49.6%) | 329 (32.7%) |  |
| **Planned enrollment** |  |  |  |  |  | < 0.001 |
| Median (Min, Max) | 259 (0, 5000) | 205 (0, 5000) | 260 (0, 3650) | 260 (0, 2750) | 366 (0, 2750) |  |
| **Cancer stage** |  |  |  |  |  | < 0.001 |
| Metastatic | 4484 (65.6%) | 1546 (71.2%) | 1475 (68.6%) | 934 (61.9%) | 529 (52.6%) |  |
| Non-Metastatic | 2120 (31.0%) | 539 (24.8%) | 612 (28.5%) | 528 (35.0%) | 441 (43.8%) |  |
| Non-specified | 236 (3.5%) | 88 (4.0%) | 63 (2.9%) | 48 (3.2%) | 37 (3.7%) |  |
